# Correlation between hyperuricemia and thickened left ventricular wall in hypertensive young adults

**DOI:** 10.1186/s12872-024-04060-1

**Published:** 2024-07-29

**Authors:** Na Wang, Weihong Lin, Xiaoli Chen, Gaojun Wu, Danhong Fang

**Affiliations:** 1 Health Care Center, The First Affiliated Hospital of Wenzhou Medical University, Nan Bai Xiang Street, Ouhai District, Wenzhou, Zhejiang 325002 China; 2 Department of Neurology, The First Affiliated Hospital of Wenzhou Medical University, Wenzhou, Zhejiang 325002 China; 3 Department of Cardiology, The First Affiliated Hospital of Wenzhou Medical University, Nan Bai Xiang Street, Ouhai District, Wenzhou, Zhejiang 325002 China

**Keywords:** Hyperuricemia, Young adult, Hypertension, Interventricular septum thickness, Left ventricular posterior wall thickness, Obesity

## Abstract

**Background:**

In this study, we examine the association between the hyperuricemia(HU) and hypertension(HTN) in Chinese young adults. Besides, the correlation between the occurrence of thickened left ventricular wall and HU was identified in patients with HTN.

**Methods:**

In all, 360 patients with HTN and 1991 young adults with normal blood pressure(NBP) were enrolled in the study. Participant characteristics were collected. Univariable and multivariable logistic regression tests were utilized to identify the correlation between the presence of HU and HTN, and the correlation between the occurrence of thickened ventricular septum and HU in patients with HTN.

**Results:**

The prevalence of HU in Chinese young adults with HTN was significantly higher than young adults with NBP(36.39% vs. 16.93%). Univariable analyses revealed that 8 factors were related with the presence of HTN with p value < 0.001, including HU, male, body mass index(BMI) ≥ 24 kg/m^2^, total cholesterol(TC) > 5.17mmol/L, triglyceride(TG) > 1.70mmol/L, high density lipoprotein cholesterol(HDL-C) < 1.0mmol/L, fasting blood glucose(FBG) > 6.10mmol/L and fatty liver. After adjusting these covariates, multivariable analysis revealed that HU[odds ratio(OR):1.47, 95% confidence interval(CI): 1.10–1.95, *p* = 0.008] remained independent association with HTN in young adults. Additionally, univariable and multivariable logistic analyses revealed that HU kept the independent effect on the presence of thickened interventricular septum(adjusted OR = 1.81, 95% CI: 1.05–3.11, *P* = 0.03) and thickened left ventricular posterior wall(adjusted OR = 2.28, 95% CI: 1.28–4.08, *P* = 0.005) in young adults with HTN.

**Conclusion:**

HU was independently associated with HTN in young adults. HU was independently correlated with thickened left ventricular wall, including interventricular septum and left ventricular posterior wall, in young adults with HTN.

**Supplementary Information:**

The online version contains supplementary material available at 10.1186/s12872-024-04060-1.

## Introduction

As one of main risk factors for cardiovascular disease, hypertension (HTN) is the first risk factor for morbidity and mortality of cardiovascular and cerebrovascular diseases in China, which is influential in public health and economic development. Although the prevalence of HTN increases by the age, the prevalence among young people is also markedly elevating in recent years as prevalence of traditional risk factors accumulates in young adults, such as obesity, diabetes mellitus (DM), renal disease, social-psychological factors and adverse living habits, like heavy drinking [1–4]. According to the Strong Heart research findings [5], compared to healthy young adults, the prevalence of thickened ventricular septum and increased left ventricular mass were remarkably higher among young adults with HTN. Harvard Alumni Health Study (HAHS) showed prospective associations between higher blood pressure in younger adults and elevated risk of all-cause mortality, cardiovascular disease (CVD), and coronary heart disease (CHD) several decades later [6]. The strong evidence about HTN risk in the young adults came from the Chicago Heart Association Detection Project in Industry study. It was proposed that younger and middle-aged adults with HTN had higher relative risk for CVD and CHD mortality, over a 31-year mean follow-up period. Additionally, the presence of HTN could be associated with the elevated mean pulse pressure, stroke volume index and total peripheral resistance index [5]. These findings demonstrated that HTN in younger adults was correlated with the long-term risk of adverse cardiovascular event.

Another evidence revealed that the high level of uric acid (UA) was correlated with relatively increasing risk of HTN, which was independent with traditional risk factors [7–15]. A meta-analysis including a total of 18 prospective cohort studies claimed that with a 1 mg/dl increase in uric acid level, the prevalence for hypertension increased by 13% [8]. The Pressioni Arteriose Monitorate E Loro Associazioni(PAMELA) Study identified that, an increase in serum uric acid of 1  mg/dl independently predicted new-onset home and ambulatory hypertension [7]. So far, there are few studies concerning relationship between HU and HTN in young adults in China.

Given that prevalence of HTN in young adults is growing worldwide, the aim of this study is to discuss the relation between HU and HTN, and the association between HU and thickened interventricular septum and left ventricular posterior wall in young adults.

## Methods

### Study population

The enrolled population was divided into 2 groups: HTN group and normal blood pressure (NBP) group. The first group included young adults (aged 18 to 45 years old) with HTN visiting the Cardiovascular Department of the First Affiliated Hospital of Wenzhou Medical University from Jan 2016 to Jan 2019. The second group included young adults, with NBP and without antihypertensives histories, having health checks in Health Care Center of the First Affiliated Hospital of Wenzhou Medical University during the same period. All past medical histories, including disease history and medicine history, were inquired and recorded. Measurements, included BP, height, weight and body mass index (BMI) were checked and recorded. Laboratory examinations, including complete blood count, blood chemistries[including UA, total cholesterol (TC), triglyceride (TG), high density lipoprotein cholesterol (HDL-C), low density lipoprotein cholesterol (LDL-C), fasting blood glucose (FBG), serum creatinine (SCr)], homocysteine and thyroid function, electrocardiogram, echocardiography and abdominal ultrasound were tested and recorded.

Excluding criteria contained (1) secondary HTN; (2) cardiovascular diseases, such as congestive heart failure, myocardiosis, coronary artery disease, valvular heart disease, congenital heart disease, and atrial fibrillation; (3) other diseases, such as thyroid dysfunction, malignancy and rheumatic immune disease.

This study was approved by institutional review board(IRB) of the First Affiliated Hospital of Wenzhou Medical University (No.2,020,107). Informed consent was waived by the institutional review board (IRB) of the First Affiliated Hospital of Wenzhou Medical University (No.2,020,107), due to cross-sectional nature of this study. All data was extracted from electronic medical records and analyzed in accordance with the principles of the Declaration of Helsinki.

### Diagnostic criteria and definition

Based on 2018 Chinese Guidelines for Prevention and Treatment of Hypertension [16], HTN diagnosis was made by clinic BP, ABPM or HBPM, excluded secondary HTN by screening, and evaluated the risk factors, target organ damages and clinical complications.

The definition of HU in the study were fasting serum UA ≥ 420 µmol/L in males and UA ≥ 360 µmol/L in females after regular purine diet [17]. BMI (kg/m^2^) was calculated by weight(kg)/height^2^(m^2^). According to Experts Consensus and standards of Chinese Overweight or Obese Weight Management, patients with BMI ≥ 24 and < 28 kg/m^2^ were considered as overweight [18]. Patients with BMI ≥ 28 kg/m^2^ were considered as obesity.

Echocardiogram was performed by ultrasonic system (Vivid E95, GE Healthcare, Amersham, UK). Based on standardized imaging protocol, parameters were measured by M-mode echocardiography and two-dimensional echocardiography, including left atrium dimension, left ventricular end diastolic dimension, interventricular septal thickness, left ventricular posterior wall thickness, aortic root dimension, ascending aorta dimension, main pulmonary artery diameter and left ventricular ejection fraction. The interventricular septal thickness and the left ventricular posterior wall thickness were measured on the same image of M-mode echocardiography. The left ventricular end diastolic dimension and the systolic dimension were obtained from left parasternal long axis imaging in mitral valve level. The left atrium dimension was measured at end-systole, namely at the time of left atrium dimension maximized. When there was no segmental wall motion abnormality, left ventricular ejection fraction was calculated by left ventricular dimension detected by left parasternal short axis view in the papillary muscle level. According to Handbook of Echocardiography by Joe K.OH, left atrium dimension > 40 mm was regarded as enlargement of left atrium; left atrium dimension ≤ 40 mm was regarded as normal left atrium. Interventricular septal thickness ≥ 11 mm was regarded as thickened ventricular septum. Interventricular septal thickness ranging from 6 to 10 mm was regarded as normal. The normal range of left ventricular posterior wall thickness was 6–10 mm, while a thickness ≥ 11 mm indicated thickening of the left ventricular posterior wall [19].

The hypertensive alteration of echocardiographic indicators was manifested as left ventricular hypertrophy (LVH), which was defined as an increase of left ventricular mass index (LVMI) above 115 g/m^2^ in males or 95 g/m^2^ in females. Left ventricular mass (LVM) was calculated as following formula: LVM = 0.8*1.04*[( interventricular septal thickness + left ventricular end diastolic dimension + left ventricular posterior wall thickness)3- left ventricular end diastolic dimension 3] + 0.6 g. LVMI (g/m^2^) was calculated by LVM(g)/ body surface area (m^2^) [19]. 

### Laboratory examinations

After 8-h fasting, the following blood parameters were measured on the morning by automatic blood cell analyzer (mindray BC6800, China), automatic biochemical analyzer (Beckman Coulter AU5800, USA) and automatic electrochemiluminescence immune-analyzer (Beckman Coulter DXI800, USA): complete blood count, blood chemistries (including UA, TC, TG, HDL-C, LDL-C, FBG, SCr), homocysteine and thyroid function.

### BP measurement

According to 2016 Canadian Hypertension Education Program Guidelines [20], BP was measured by specifically trained medical professions. Office BP measurement was taken with a non-automated device (non-AOBP) or an automated device (AOBP, HEM-705CP, Omron, Kyoto, Japan). After emptying the bladder and taking a rest in quiet-environment for 5 min, subjects took BP measurements. In the course of the rest and measurement, subjects and medical professions kept silence. Two or three times-repeats of measurement were conducted and time interval for each measurement was 1 to 2 min. Only when the difference between the first two measurements is greater than 10 mmHg, subjects were measured for the third time, and the average of the last two times was recorded for each data. For most subjects, standard cuffs (size 13 cm*35 cm) were selected. For subjects with big or small arm circumferences, appropriate cuffs were selected. When BP measurement was taken by a non-AOBP, Korotkoff sounds in phase I and V (sudden reduction or disappearence of auscultation sound) were used to recognize the SBP and DBP respectively. The BP of both arms was measured at the first clinic visit to detect the possible BP difference between the arms. Then the arm with higher BP was recorded as the BP reference.

### Statistical analysis

Statistical analyses were performed using SPSS 19.0 version. All analyses were two sided, and a P value < 0.05 was considered statistically significant. Continuous variables obeying normal distribution were presented as means ± standard deviation (SD) and compared by Student t test. Continuous variables not obeying normal distribution were presented as medians (ranges) and compared by Mann-Whitney U test. Categorical variables were presented as frequencies (percentages) and compared by χ^2^ tests or Fisher test. The association between two variables were assessed by Pearson correlation analysis. Univariate and multivariate logistic regression models were used to evaluate the effects of HU on the risk of HTN and thickened ventricular septum. The covariates with P value < 0.1 in the univariate logistic regression analyses were included in the multivariate logistic regression models. Additionally, covariates with a great clinical importance were included in the multivariate logistic regression.

## Results

### Participant characteristic

The comparison of demographic, clinical characteristics, and cardiac indicators between the young adults with NBP and the HTN was shown in Table 1. The average age of the HTN group (*n* = 360 cases) was 38.78 (± 5.62) years old, while the average age of the NBP group (*n* = 1991 cases) was 38.17 (± 5.46) years old. There was no significant difference in age between the two groups (*P* > 0.05). The proportions of HU and fatty liver in HTN group were significantly higher than those in NBP group (*P* < 0.01). The BMI, TC, TG, LDL-C, UA, FPG, creatinine, and homocysteine levels in HTN group were significantly higher than those in NBP group (*P* < 0.001). The level of HDL-C was significantly lower than that of the NBP group (*P* < 0.001). Compared with the NBP group, various cardiac parameters(aortic root diameter, inner diameter of ascending aorta, left ventricular end diastolic diameter, left ventricular end systolic diameter, interventricular septal thickness, left ventricular posterior wall thickness, left atrial diameter, main pulmonary artery diameter, stroke output, and cardiac output) in the HTN group were significantly increased (*P* < 0.001). However, there was no significant difference between the two groups in left ventricular shortening fraction (*P* = 0.300) and left ventricular ejection fraction (*P* = 0.133). Besides, in order to correct for confounding factor, sex, the stratified analysis for cardiac parameters was displayed in Supplementary Table 1.

**Table 1:** Comparison of demographic and clinical characteristics between young adults with HTN and normal BP

Variables	HTN group(*n* = 360)	NBP group(*n* = 1991)	*P* value
Sex, male, n(%)	310(86.11)	1190(59.77)	<0.001
Age(years)	38.78 ± 5.62	38.17 ± 5.46	0.054
HU, n(%)	131(36.39)	337(16.93)	<0.001
Fatty liver, n(%)	151(41.94)	326(16.37)	<0.001
BMI (kg/m2)	25.66 ± 3.50	23.04 ± 3.30	<0.001
SBP (mmHg)	151.57 ± 11.91	115.82 ± 12.18	<0.001
DBP (mmHg)	93.92 ± 13.11	68.79 ± 9.77	<0.001
UA (µmol/L)	394.92 ± 98.37	329.28 ± 88.10	<0.001
TC (mmol/L)	5.39 ± 1.35	5.12 ± 1.07	<0.001
TG (mmol/L)	1.89(1.18,2.81)	1.27(0.87,1.97)	<0.001
HDL-C (mmol/L)	1.17 ± 0.26	1.34 ± 0.33	<0.001
LDL-C (mmol/L)	3.18 ± 0.84	3.03 ± 0.83	0.002
FPG (mmol/L)	5.06 ± 1.12	4.67 ± 1.02	<0.001
Creatinine (µmol/L)	69.64 ± 13.14	64.93 ± 13.73	<0.001
Homocysteine (µmol/L)	10.82 ± 5.83	8.91 ± 4.49	<0.001
Echocardiographic indicators			
Aortic root diameter(mm)	34(26–43)	32(22–44)	<0.001
Inner diameter of ascending aorta(mm)	32(25–42)	30(21–41)	<0.001
Left ventricular end diastolic diameter(mm)	48(39–65)	47(35–61)	<0.001
Left ventricular end systolic diameter(mm)	30(23–54)	30(20–43)	<0.001
Left atrial diameter(mm)	38(29–58)	35(23–48)	<0.001
Interventricular septum thickness(mm)	10(7–13)	9(6–17)	<0.001
Left ventricular posterior wall thickness(mm)	10(7–12)	9(6–12)	<0.001
Main pulmonary artery diameter(mm)	23(18–34)	22(16–32)	<0.001
Stroke output(ml)	72.5(45.7-122.6)	67.3(32.8-114.3)	<0.001
Left ventricular shortening fraction(%)	36(17–49)	37(26–51)	0.300
Left ventricular ejection fraction(%)	65.9(34.6–80.0)	66.2(51.3–82.3)	0.133

*Notes* BMI: body mass index, SBP: systolic pressure, DBP: diastolic pressure, UA: serum uric acid, TC: total cholesterol, TG: triglyceride, HDL-C: high density lipoprotein cholesterol, LDL-C: low density lipoprotein cholesterol, FPG: Fasting plasma glucose

### Characteristics and associated factors of HTN in young adults

**Fig. 1:**
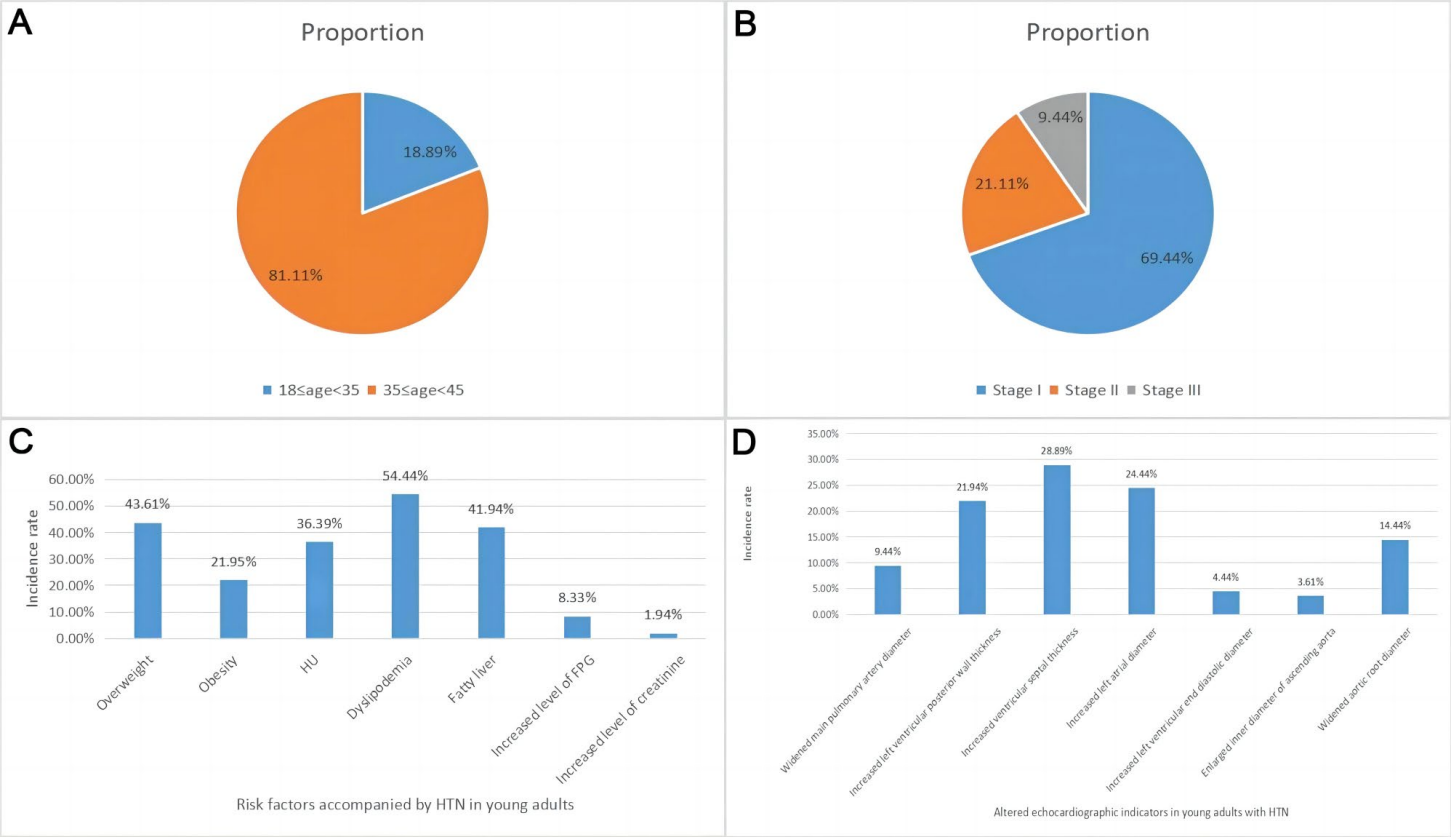
Characteristics and associated factors of HTN in young adults. **A**: The proportion of different age stratification in young adults with HTN; **B**: The proportion of different HTN grade in young adults with HTN; **C**: The prevalence rate of associated factors accompanied by HTN in young adults; **D**: The prevalence rate of altered echocardiographic indicators in young adults with HTN

The characteristics and associated factors of HTN in young adults are shown in Table 1; Fig. 1. In young adults, 86.11% of patients with HTN are male, much higher than female patients (13.89%). The vast majority of patients with HTN are aged between 35 and 45 years (81.11%). More than two-thirds of the patients (69.44%) had grade 1 HTN, 21.11% had grade 2 HTN, and 9.44% had grade 3 HTN. Nearly two-thirds of patients are overweight and obese, with obese patients accounting for 21.94%. Over half of patients with dyslipidemia, 36.39% with HU, and 41.94% with fatty liver. The 8.33% of HTN patients had elevated fasting plasma glucose, while only 1.94% of patients had a slight increase in serum creatinine. Nearly 1/3 of the patients had cardiac pathological changes, and the ventricular septal thickness was the most susceptible, with about 28.89% patients having thickened ventricular septal. Additionally, 24.44% patients had increased left atrial inner diameter, 21.94% patients had thickened left ventricular posterior wall, 14.44% patients had widened aortic root inner diameter, 9.44% patients had widened main pulmonary artery inner diameter, 4.44% patients had increased left ventricular end diastolic inner diameter, and 3.61% patients had widened ascending aorta inner diameter.

### Association of HU and HTN in young adults

Table 2 indicates that showed that factors, like male, old age, high BMI, HU, dyslipidemia (including high TC levels, high TG levels, low HDL-C levels and high LDL-C levels), elevated FPG, elevated creatinine and fatty liver, were significantly correlated with HTN in the young-aged population (*P* < 0.05) by univariate logistic regression analysis. Incorporating the above clinical and biochemical covariates into a multivariate logistic regression model, the results showed an independent correlation between HU and HTN (adjusted OR = 1.47, 95% CI: 1.10–1.95, *P* = 0.008) in young adults. In addition, HTN in young adults was independently associated with male (adjusted OR = 2.44, 95% CI: 1.72–3.45, *P* < 0.001), high BMI (adjusted OR = 1.69, 95% CI: 1.28–2.25, *P* < 0.001), elevated FPG (adjusted OR = 2.13, 95% CI: 1.28–3.55, *P* = 0.004), and fatty liver (adjusted OR = 1.75, 95% CI: 1.30–2.35, *P* < 0.001) (Table 2).

**Table 2:** Univariable and multivariable logistic regression analyses for hypertension in young adults

Variables	Univariable analysis		Multivariable analysis	
	Unadjusted OR (95% CI)	*P* value	Adjusted OR (95% CI)	*P* value
Sex, male vs. female	4.17(3.03–5.56)	<0.001	2.44(1.72–3.45)	<0.001
Age(years)	1.02(1.00-1.05)	0.031	1.02(1.00-1.05)	0.08
BMI(kg/m2), ≥ 24vs < 24	3.23(2.55–4.10)	<0.001	1.69(1.28–2.25)	<0.001
HU, yes vs. no	2.81(2.20–3.59)	<0.001	1.47(1.10–1.95)	0.008
TC*(mmol/L), > 5.17vs ≤ 5.17	1.62(1.29–2.03)	<0.001	1.17(0.82–1.67)	0.396
TG* (mmol/L),>1.70vs ≤ 1.70	2.58(2.05–3.25)	<0.001	1.00(0.74–1.33)	0.971
HDL-C# (mmol/L), < 1.0vs ≥ 1.0	2.63(2.02–3.41)	<0.001	1.35(1.00-1.84)	0.051
LDL-C* (mmol/L), > 3.10vs ≤ 3.10	1.44(1.15–1.81)	0.002	0.97(0.69–1.37)	0.878
FPG* (mmol/L), > 6.10vs ≤ 6.10	3.79(2.36–6.08)	<0.001	2.13(1.28–3.55)	0.004
Creatinine *(µmol/L), Male, > 97vs ≤ 97, Female, > 80vs ≤ 80	2.61(1.06–6.45)	0.037	1.36(0.49–3.75)	0.554
Fat liver, yes vs. no	3.69(2.90–4.69)	<0.001	1.75(1.30–2.35)	<0.001

*Notes* OR: odds ratio, CI: confidence interval, HU: hyperuricemia, BMI: body mass index, TC: total cholesterol, TG: triglyceride, HDL-C: high-density lipoprotein cholesterol, LDL-C: low-density lipoprotein cholesterol, FPG: fasting plasma glucose. *: Refer to the upper limit of our hospital’s inspection items; #: Refer to the 2018 China Guidelines for the Prevention and Treatment of Hypertension (Cardiovascular Risk Factors)

*The relationship between HU and* t*hickened interventricular septum and left ventricular posterior wall in young adults with HTN*.

Furthermore, the associated factors for interventricular septum and left ventricular posterior wall thickenings in hypertensive young adults were analyzed in this study.

In the HTN group, univariate logistic regression analysis showed that factors, such as male, HU, high SBP and DBP, obesity, hypertriglyceridemia and low level of HDL-C were significantly correlated with interventricular septum thickening (*P* < 0.01). As for left ventricular posterior wall thickening, apart from these factors, age was an associated factor (*P* < 0.1) by univariate analysis. The multivariate logistic regression results showed that in the HTN group, HU was independently related to ventricular septal thickening(adjusted OR = 1.81, 95% CI: 1.05–3.11, *P* = 0.03) and left ventricular posterior wall thickening(adjusted OR = 2.28, 95% CI: 1.28–4.08, *P* = 0.005) (see Tables 3 and 4). It suggests that HU is independently associated with thickened left ventricular wall in young adults with HTN.

Besides, in NBP group, there were no similar associations discovered between HU and the thickened interventricular septum and left ventricular posterior wall, with *P* > 0.05 (Supplementary Tables 2 and 3).

Furthermore, in our study, only 32 (8.9%) participants were diagnosed with LVH. And subsequent univariable logistic progression analysis result showed that OR of hyperuricemia for LVH was 0.95 (95%CI: 0.43–2.01, *p* = 0.90).

**Table 3:** Univariate and multivariate logistic regression analyses for thickened interventricular septum in young adults with hypertension

Variables	Univariable analysis		Multiple analysis	
	Unadjusted OR(95% CI)	P value	Adjusted OR(95% CI)	P value
Age(years)	1.04(0.99–1.08)	0.109	1.08(1.03–1.14)	0.003
Sex, female vs. male	0.13(0.03–0.36)	<0.001	0.12(0.02–0.44)	0.006
BMI(kg/m^2^), ≥ 28 vs. < 28	4.01(2.36–6.83)	<0.001	3.5(1.95–6.34)	<0.001
SBP (mmHg)	1.03(1.01–1.05)	0.001	1.01(0.98–1.04)	0.503
DBP (mmHg)	1.04(1.03–1.06)	<0.001	1.04(1.02–1.06)	<0.001
HU, Yes vs. No	2.09(1.30–3.34)	0.002	1.81(1.05–3.11)	0.032
TC^*^(mmol/L), > 5.17vs ≤ 5.17	1.44(0.90–2.30)	0.129		
TG^*^ (mmol/L),>1.70vs ≤ 1.70	2.43(1.49–3.96)	<0.001	1.36(0.76–2.42)	0.299
HDL-C^#^ (mmol/L), < 1.0vs ≥ 1.0	2.07(1.26–3.40)	0.004	1.79(1.01–3.18)	0.045
LDL-C^*^ (mmol/L), > 3.10vs ≤ 3.10	1.29(0.81–2.05)	0.279		
FPG^*^ (mmol/L), > 6.10vs ≤ 6.10	1.33(0.59–2.99)	0.490		
Creatinine*(µmol/L),				
Male, > 97 vs. ≤ 97,	0.78(0.08–7.59)	0.830		
Female, > 80 vs. ≤ 80				

*Notes* OR: odds ratio, CI: confidence interval, HU: hyperuricemia, BMI: body mass index, SBP: systolic pressure, DBP: diastolic pressure, TC: total cholesterol, TG: triglyceride, HDL-C: high-density lipoprotein cholesterol, LDL-C: low-density lipoprotein cholesterol, FPG: fasting plasma glucose. *: Refer to the upper limit of our hospital’s inspection items; #: Refer to the 2018 China Guidelines for the Prevention and Treatment of Hypertension (Cardiovascular Risk Factors)

**Table 4:** Univariate and multivariate logistic regression analyses for the thickened left ventricular posterior wall in young adults with hypertension

Variables	Univariable analysis		Multiple analysis	
	Unadjusted OR(95% CI)	*P* value	Adjusted OR(95% CI)	*P* value
Age(years)	1.05(1.00-1.10)	0.070	1.09(1.03–1.16)	0.004
Sex, female vs. male	0.19(0.05–0.54)	0.007	0.28(0.06–0.88)	0.051
BMI(kg/m^2^), ≥ 28 vs. < 28	3.39(1.95–5.89)	<0.001	3.22(1.75–5.96)	<0.001
SBP(mmHg)	1.04(1.02–1.06)	<0.001	1.02(0.99–1.05)	0.25
DBP(mmHg)	1.05(1.03–1.08)	<0.001	1.05(1.03–1.08)	<0.001
HU, yes vs. no	2.38(1.43–3.96)	0.001	2.28(1.28–4.08)	0.005
TC^*^(mmol/L), > 5.17vs ≤ 5.17	1.33(0.79–2.21)	0.282		
TG^*^ (mmol/L), > 1.70vs ≤ 1.70	2.12(1.25–3.61)	0.006	1.20(0.64–2.27)	0.568
HDL-C^#^ (mmol/L), < 1.0vs ≥ 1.0	1.88(1.10–3.20)	0.021	1.59(0.85–2.95)	0.141
LDL-C^*^ (mmol/L), > 3.10vs ≤ 3.10	1.33(0.80–2.22)	0.267		
FPG^*^ (mmol/L), > 6.10vs ≤ 6.10	1.40(0.59–3.31)	0.447		
Creatinine ^*^(µmol/L),				
Male,>97vs ≤ 97,	1.14(0.12–11.08)	0.912		
Female > 80vs ≤ 80				

*Notes* OR: odds ratio, CI: confidence interval, HU: hyperuricemia, BMI: body mass index, SBP: systolic pressure, DBP: diastolic pressure, TC: total cholesterol, TG: triglyceride, HDL-C: high-density lipoprotein cholesterol, LDL-C: low-density lipoprotein cholesterol, FPG: fasting plasma glucose. *:Refer to the upper limit of our hospital’s inspection items; #: Refer to the 2018 China Guidelines for the Prevention and Treatment of Hypertension (Cardiovascular Risk Factors)

## Discussion

Our research shows that: (1) HU is one of the main associated factors for hypertension in young adults, while other associated factors include high BMI, dyslipidemia, and fatty liver; (2) Nearly 1/3 of young hypertensive patients have cardiac pathological changes. The most vulnerable part is the thickness of the interventricular septum, followed by the diameter of the left atrium and the thickness of the left ventricular posterior wall; and (3) HU is independently associated with thickened left ventricular wall, including thickened interventricular septum and left ventricular posterior wall, in young adults with HTN. In view of the fact that the prevalence of HTN is on the rise among young adults, it is attributed to the increase of combined associated factors for young adults, such as obesity, diabetes mellitus (DM), renal disease, social-psychological factors [1–4, 21]. Therefore, in order to better prevent and treat HTN in young adults, we explored the correlation between HTN and another associated factor, HU, in young adults, and further explored the relationship between HU and left ventricular wall in HTN patients.

In recent years, attention has gradually begun to be paid to hypertension in young adults both domestically and internationally. Paul GK et al [21]. conducted a cross-sectional descriptive observational study on 322 young people with HTN in Bangladesh, and found that the main risk factors for HTN in young adults were smoking, obesity, abnormal blood lipids, high salt intake and the use of chewing tobacco. The 11.5% of patients had elevated level of serum creatinine, and 18.6% of patients experienced concentric LVH. The common comorbidity was ischemic heart disease and diabetes. Ondimu DO et al. [4] conducted a case-control study on young people aged 18–35 in Bomet County, Kenya. The results showed that individuals with factors, such as overweight, family history, alcohol consumption, had an increased risk of developing HTN. This study conducted a cross-sectional observational study on Chinese young adults aged 18 to 45 years old. The results suggested that HU was an independent associated factor for HTN in young adults. The correlation between elevated serum UA level and HTN had been reported in the past [7–15]. Therefore, it was consistent with the results of this study.

Previous Strong Heart studies investigated the effects of HTN on heart and hemodynamics in subjects aged 14 to 39 [5], and found that compared to young people with NBP, HTN patients had a higher incidence of left ventricular wall thickening, left ventricular mass increasing, relative wall thickening and LVH. There were also studies suggesting that hypertension was associated with higher average pulse pressure, stroke volume index, and total peripheral resistance index [22]. Consistent with these research results, this study suggested that nearly one-third of young and middle-aged patients with HTN exhibited cardiac pathological changes. Compared with the young adults with NBP, the HTN group had significantly increased cardiac indexes, including left atrial diameter, interventricular septum thickness, left ventricular posterior wall thickness, left ventricular end diastolic diameter, left ventricular end systolic diameter, aortic root diameter, ascending aorta diameter, main pulmonary artery diameter, stroke volume and cardiac output (all *P* < 0.001). Among these cardiac indexes, the thickness of the interventricular septum is the most likely to be involved in heart target organ damage in young adults with HTN, followed by the diameter of the left atrium and the thickness of the left ventricular posterior wall.

As we all know, the cardiac pathological changes of HTN are mainly LVH, which is manifested in the increase of left ventricular mass index (LVMI), interventricular septum hypertrophy and left ventricular posterior wall thickening on echocardiography. In this study, we further explored the relationship between HU and thickened left ventricular wall in young adults with HTN. The results suggested that HU was independently related to ventricular septal thickening and left ventricular posterior wall thickening. It was indicated that HU was an independent associated factor for thickened left ventricular wall. Previous studies have suggested that serum UA levels are associated with LVH and diastolic dysfunction in female patients with preserved ejection fraction heart failure [23]. Another study has reported an independent association between UA levels and LVMI [24]. These studies involved the relationship between serum UA levels and LVH, LVMI, while the correlation between HU and LVH in young adults with HTN has not been explored. Therefore, this study supplemented previous research findings and further revealed the relationship between HU and thickened left ventricular wall in young adults with HTN.

Our study suggests that, in addition to HU, obesity was also an independent associated factor for interventricular septum thickening and left ventricular posterior wall thickening in young adults with HTN. When HTN in young adults was combined with multiple associated factors, especially when combined with HU and obesity, it would further promote LVH. Therefore, it was necessary to perform routine echocardiography on these young HTN patients. It was advocated that early monitoring, treatment, actively management of associated factors such as HU and obesity, and combination of antihypertensive therapy could improve or even reverse LVH and prevent future cardiovascular events.

Besides, in our study, it was indicated that DBP was an associated factor for thickened interventricular septum and left ventricular posterior wall, rather than SBP. As a previous study proposed that elevated SBP had often been an innocent clinical condition in physically active young people, which caused by an elevated pulse pressure amplification and low wave arterial reflection from peripheral sites caused by increased arterial elasticity [25]. Additionally, hypertensive patients in young adults mostly showed elevated DBP. A summary analysis of 489,814 participants in 15 cohort studies (including 87,346 participants from the Kailuan prospective cohort study in China) found that elevated DBP significantly increased the risk of stroke, cardiovascular death, and composite cardiovascular events compared to normal blood pressure, and significantly increased the risk of composite cardiovascular events in Asian and young adults under 55 years old with elevated DBP [26]. Consistent with these research findings, our study discovered that elevated DBP was associated with thickened interventricular septum and left ventricular posterior wall.

The results of this study will enrich the knowledge of clinical doctors and policy makers. When formulating preventive measures, various main associated factors for HTN in young adults should be considered to ensure better preventive health services for young adults at risk of HTN. In the coming decades, as young people transition to old age today, there will be potential opportunities to reduce the burden of future cardiovascular diseases and promote cardiovascular health.

There were still some potential limitations in this study. First of all, due to the lack of detailed family history, personal history (smoking and drinking), diet habits, education level and other data in this study, there was no observational description of relevant data. Further data is needed to comprehensively evaluate the risk factors for HTN in young adults. Second, the subjects included in this study were mainly from the medical and physical examination personnel of the First Affiliated Hospital of Wenzhou Medical University, and there was a selection bias, so it is necessary to conduct a multi-center study and increase the number of cases to reduce selection bias in the further study. Third, there were fewer female cases in this study, which might be related to the existence of selection bias, so it is necessary to sample in a larger HTN database to reduce this bias. At the same time, gender grouping analysis could also be conducted to eliminate gender interference. Fourthly, LVMI is of the superior clinical and prognostic value, compared to the thickness of the interventricular septum and left ventricular posterior wall. Unfortunately, in our study, only 32 (8.9%) participants were diagnosed with LVH, based on LVMI which calculated according to 2018 Chinese Guidelines for Prevention and Treatment of Hypertension [16]. And subsequent univariable logistic progression analysis result showed that OR of hyperuricemia for LVH was 0.95(95%CI: 0.43–2.01, *p* = 0.90). It might be due to the inadequate sample size and restrictive time of suffering from the HTN for young adults. Hypertensive left ventricular wall thickening is an early manifestation of LVH. Although it has not yet reached the level of LVH, ventricular remodeling has begun to occur. So, the alterations in echocardiographic phenotype are only restricted to interventricular septum and left ventricular posterior wall thickness, but the incidence of LVH was insufficient. Finally, it was a cross-sectional study, therefore can only find associations, but no causation. Further prospective research is needed to determine whether combined UA lowering therapy can prevent cardiovascular disease and improve cardiovascular prognosis.

## Conclusion

Our research indicates that HU is one of the non-traditional main associated factors for HTN in young adults, while other associated factors include overweight, obesity, dyslipidemia, and fatty liver. HU is independently associated with thickened left ventricular wall in young adults with HTN.

### Electronic supplementary material

Below is the link to the electronic supplementary material.

Supplementary Material 1

Supplementary Material 2

Supplementary Material 3

## Data Availability

The data could be acquired from the corresponding author on reasonable request.
